# Hyper-O-GlcNAcylation promotes epithelial-mesenchymal transition in endometrial cancer cells

**DOI:** 10.18632/oncotarget.26884

**Published:** 2019-04-23

**Authors:** Nicole Morin Jaskiewicz, David H. Townson

**Affiliations:** ^1^Department of Molecular, Cellular and Biomedical Sciences, University of New Hampshire, Durham, NH, USA; ^2^Department of Animal and Veterinary Sciences, University of Vermont, Burlington, VT, USA

**Keywords:** O-GlcNAc, epithelial-mesenchymal transition, endometrial cancer, metastasis, diabetes

## Abstract

Diabetic women have a 2–3 fold increased risk of developing endometrial cancer, however, the molecular aspects of this risk are not fully understood. This study investigated the alteration of cellular O-GlcNAcylation of proteins as the potential mechanistic connection between these two conditions. The endometrial cancer cell line (Ishikawa) was utilized to study the effect of dysregulation of O-GlcNAcylation on epithelial mesenchymal transition (EMT). Hyper-O-GlcNAcylation (via 1 μM Thiamet-G/ThmG or 25 mM Glucose) enhanced the expression of EMT-associated genes (*WNT5B* and *FOXC2*), and protein expression of the EMT adhesion molecule, N-Cadherin. Reorganization of stress filaments (actin filaments), consistent with EMT, was also noted in ThmG-treated cells. Interestingly, Hypo-O-GlcNAcylation (via 50 μM OSMI-1) also upregulated *WNT5B*, inferring that any disruption to O-GlcNAc cycling impacts EMT. However, Hypo-O-GlcNAcylation reduced overall cellular proliferation/migration and the expression of pro-EMT genes (*AHNAK, TGFB2, FGFBP1, CALD1, TFPI2*). In summary, disruption of O-GlcNAc cycling (i.e., Hyper- or Hypo-O-GlcNAcylation) promoted EMT at both the molecular and cellular levels, but only Hyper-O-GlcNAcylation provoked cellular proliferation/migration, and cytoskeletal reorganization.

## INTRODUCTION

β-N-acetylglucosaminylation (O-GlcNAcylation) is a unique form of glycosylation that occurs on serine and threonine residues of proteins throughout the cytoplasmic and nuclear compartment of cells. It is a monosaccharide that is dynamically cycled akin to that of O-phosphorylation. The enzyme O-GlcNAc transferase (OGT) adds O-GlcNAc to proteins via the substrate UDP-GlcNAc, while O-GlcNAcase (OGA) is responsible for its removal. O-GlcNAc influences the extent of O-phosphorylation of proteins through shared binding sites and steric hindrance [1]. As the end-product of the Hexosamine Biosynthesis pathway (HBP), UDP-GlcNAc and subsequent O-GlcNAcylation is considered a nutrient sensor to the overall metabolic status of the cell. Two to five percent of the glucose that enters the cell is channeled into the HBP. As such, it is not surprising that increased O-GlcNAcylation is implicated in the development of insulin resistance [2]. Aberrant O-GlcNAcylation is a characteristic of heart disease, neurodegenerative disorders such as Alzheimer’s disease, and is a hallmark of many cancers, including endometrial cancer [3]. A relationship between glycosylation and metastasis is also evident in lung cancers, wherein epithelial-mesenchymal transition (EMT) acts through the HBP as an inducer of aberrant glycosylation [4]. Despite these observations, very little is known about the mechanistic actions of O-GlcNAcylation in cancer, specifically, in the EMT process.

Many illnesses associated with aberrant O-GlcNAcylation are also co-morbidities of Type 2 Diabetes (T2D). For instance, women with T2D have a 2-fold greater risk of developing endometrial cancer than their healthy cohorts [5]. While the incidence of many forms of cancer is declining, endometrial cancer remains among those increasing for all women [6]. Endometrial cancer results from the abnormal growth, migration, and invasion of cells that line the uterus. Common genetic alterations in endometrial tumors include *PTEN*, *PIK3CA*, *CTNNB1* (beta-catenin), and *KRAS*, all of which are related to metabolism [7]. Moreover, all of these genes influence epithelial-mesenchymal transition (EMT) signaling pathways, and recent studies indicate that the E-cadherin repressors *Slug*, *ZEB1*, and *HMGA2* are preferentially expressed along the myometrial invasive edge of tumors [8]. Snail1, a key regulator of EMT, is stabilized by O-GlcNAcylation in several cell types [9], and the mRNA of O-GlcNAc cycling enzymes (OGT and OGA) is up-regulated in endometrial tumors [3], suggesting that O-GlcNAcylation influences metastasis.

The gold standard for treatment of endometrial cancer is radiation therapy and surgery; however, 5–30% of women with endometrial cancer are premenopausal and under the age of 50 at the time of diagnosis. For these women, fertility-sparing treatments, such as progestin therapy, are an option [10]. A recent meta-analysis determined that women treated with hormonal therapy methods had a pooled regression rate of 76.2%, with 28% live births reported; however, a 40.6% relapse rate was also noted [11]. These findings underscore the importance of identifying basic mechanisms by which metabolism and O-GlcNAcylation influence the progression of endometrial cancer, with the goal of improving fertility-sparing treatments. The objective of the current study was to determine some of these mechanisms, specifically focusing on the manipulation of O-GlcNAc cycling enzymes (OGT and OGA) and their impact on molecular and cellular aspects of Epithelial-Mesenchymal Transition (EMT).

## RESULTS

### The O-GlcNAc cycling enzymes, *OGT and OGA*, are altered in endometrial cancer

**Figure 1:**
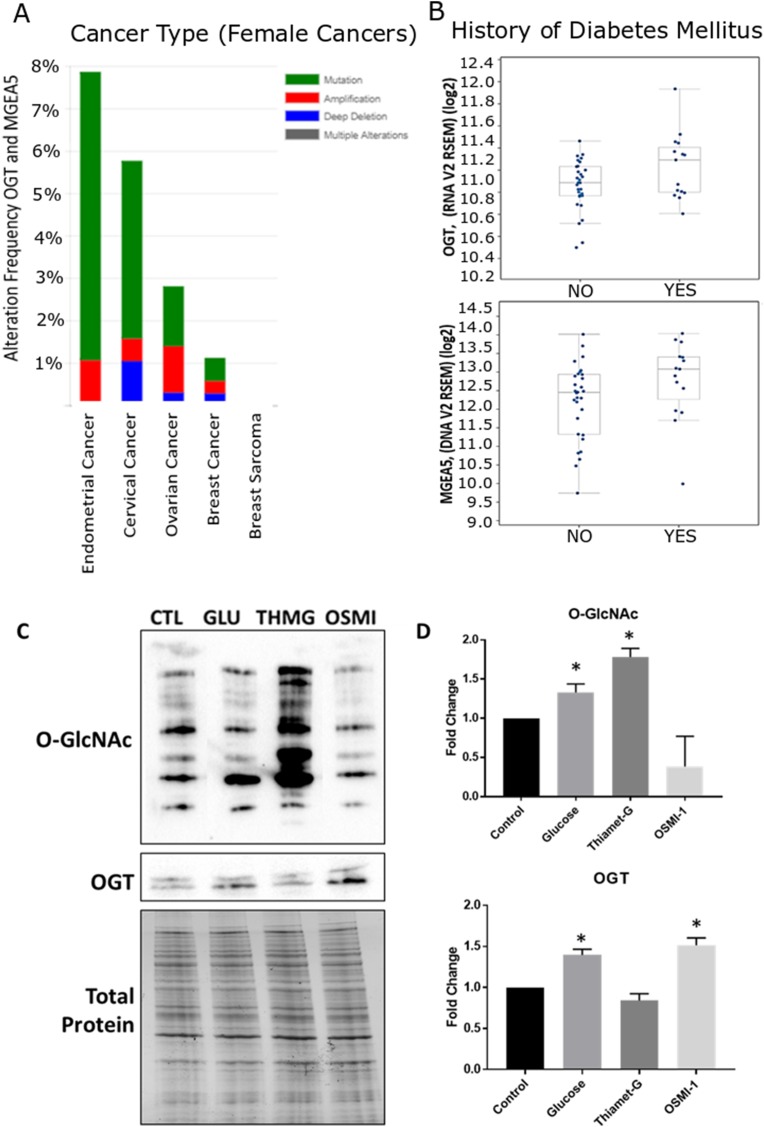
Meta-analysis of O-GlcNAc alteration in female cancers and validation of global O-GlcNAc modification in Ishikawa cells. Cancer genomics data analysis depicting a cross-cancer O-GlcNAc enzyme (OGT, MGEA5) alteration summary of female cancers. (**A**) Histograms depicting the level of gene amplification, mutation, or deletion in each data set. Data was mined from the TCGA database and was analyzed with the cBioPortal web analysis tool. Of these 18 female cancer datasets, uterine/endometrial cancer ranked the highest for gene alterations of OGT and MGEA5. (**B**) Box plots comparing the relative mRNA expression of OGT and MGEA5 in patients with or without a diagnosis of diabetes mellitus (DM). Additional analysis of the data set revealed that OGT and MGEA5 mRNA were more highly expressed in endometrial cancer patients with DM. (**C**) Western Blot analysis of global O-GlcNAc modification and OGT expression, relative to total protein, in whole cell Ishikawa lysates treated for 24 hours with the OGT inhibitor, OSMI-1; the OGA inhibitor, Thiamet-G; or supplemented with 25 mM Glucose. (**D**) Densitometry analysis of Western Blots, bars represent the mean +/– SEM (*n* = 4), (^*^) denotes statistically significant differences in density compared to control (*p* < 0.05).

Analysis of gene alterations of OGT and OGA (*OGT* and *MGEA5*, respectively) using data from the RNAseq and Microarray databases available in cBioPortal (http://cbioportal.org) revealed that of the 18 female reproductive cancer databases available, Uterine/Endometrial Cancer ranks highest for gene alterations to *OGT* and *MGEA5*, including both mutational and amplification modifications (Figure 1A). Additionally, both genes are upregulated in screened participants with Diabetes Mellitus compared to non-Diabetic controls (Figure 1B).

### Detection and manipulation of O-GlcNAcylation in the endometrial cancer line, Ishikawa

Immunodetection of global O-GlcNAcylation in Ishikawa cells revealed this form of protein modification was upregulated (Hyper-O-GlcNAcylation) in cells by supplementing complete media with 25 mM Glucose or by inhibiting OGA with Thiamet-G (1 μM; ThmG; *p* < 0.05; Figure 1C and 1D). While a qualitative decrease in O-GlcNAc expression was noted by inhibiting OGT with OSMI-1 (50 μM; OSMI-1), relative expression did not differ from controls (*p* > 0.05; Figure 1C and 1D). Interestingly, however, high glucose and OGT inhibition each enhanced relative OGT expression (*p* < 0.05; Figure 1C and 1D). In all subsequent experiments, these same manipulations of O-GlcNAcylation were utilized to determine effects of aberrant O-GlcNAcylation on phenotypic changes in Ishikawa cells (i.e., cell proliferation/migration and invasion), as well as morphological and molecular parameters associated with EMT.

### Hyper-O-GlcNAcylation supports endometrial cancer cell proliferation/migration, and promotes invasion

**Figure 2:**
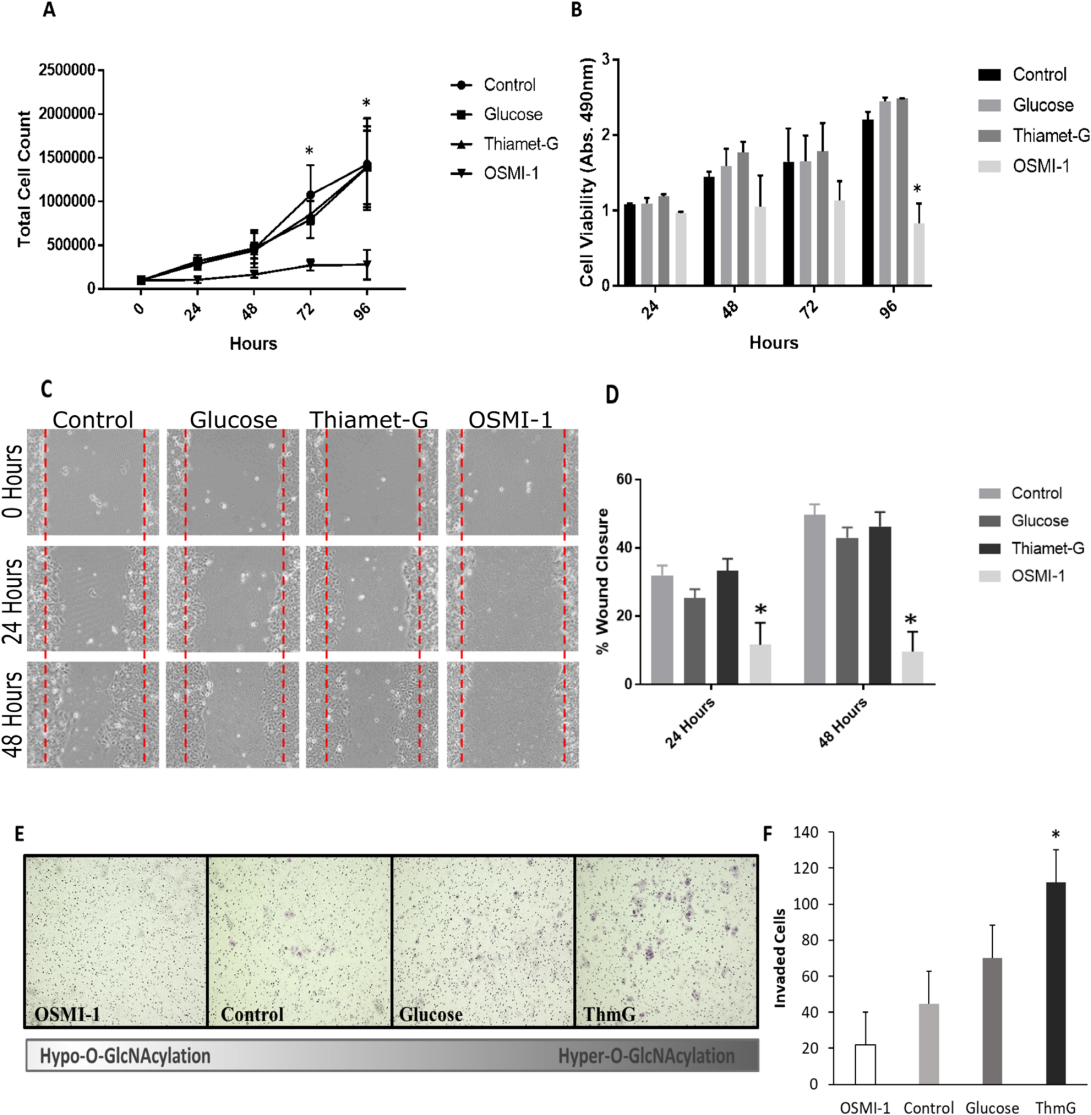
O-GlcNAcylation is necessary for Ishikawa cell proliferation and migration. (**A**) Cell growth curve depicting cell proliferation over 96 hours under serum free conditions in cells exposed to 25 mM Glucose, Thiamet-G, OSMI-1, or vehicle (media refreshed every 24 hours). Each point in the graph represents the mean +/– SEM of 3 biological replicates. An asterisk (^*^) indicates a difference between OSMI-1 treated cells and all other treatment groups (*p* < 0.05). (**B**) Bar graph representing the mean absorbance (*n* = 3) +/– SEM of MTS cell viability/proliferation assays. OSMI-1 treated cells did not proliferate, but cell viability was maintained throughout the culture. An asterisk (^*^) indicates a difference between OSMI-1 treated cells and all other treatment groups (*p* < 0.05). (**C**) Representative images of a wound healing assay evaluating the effects of Thiamet-G, Glucose, OSMI-1, or vehicle on migration of Ishikawa cells in serum free conditions. “Wounds” were imaged every 24 hours for 48 hours (100X). (**D**) Bar graphs of the wound healing assay. Mean +/– SEM (*n* = 3) of the percent of wound closure depicted. An asterisk (^*^) indicates a difference between OSMI-1 treated cells and all other treatment groups (*p* < 0.05). (**E**) Representative images of invasive cells following a Biocoat Matrigel Transwell Invasion assay (100X). Purple foci depict invasive cells. (**F**) Bar graph of the invasion assay depictin the mean +/– SEM (*n* = 4) of invaded cells after 48 hours of culture. An asterisk (^*^) indicates a difference between ThmG treated cells compared to Control (*p* < 0.05).

Cell proliferation in response to altered O-GlcNAcylation was assessed via growth curve and MTS assay in serum free conditions (Figure 2A and 2B). Ishikawa cells proliferated in serum free conditions throughout 96 hours of culture, however, inhibition of OGT (OSMI-1) impaired proliferation beginning at 72 hours compared to control and OGA-inhibited (Thiamet-G), hyper-O-GlcNAcylated cells. Similar results were observed in MTS assays. Inhibition of proliferation occurred in OGT-inhibited (OSMI-1) cells compared to all other treatment groups between 72 and 96 hours of culture (*p* < 0.05, Figure 2B), but cell viability was unchanged in this group during the entire 96 hour culture period (Figure 2A and 2B).

Wound healing assays demonstrated that Hyper-O-GlcNAcylation supported Ishikawa cell migration, with no difference in wound closure observed among Control, Glucose and ThmG-treated cultures (*p* > 0.05; Figure 2C and 2D). Hence, Hyper-O-GlcNAcylation was conducive to wound closure. Conversely, Hypo-O-GlcNAcylation (via OSMI-1) impaired cell migration (*p* < 0.05), resulting in ~10% wound closure after 48 hours of culture, compared to ~45% in Control and Hyper-O-GlcNAcylated cells (Figure 2C and 2D).

Although Ishikawa cells are considered relatively-low metastatic cells [12], Hyper-O-GlcNAcylation (i.e., ThmG treatment) augmented invasiveness compared to Control and Hypo-O-GlcNAcylated (OSMI-1-treated) cells (*p* < 0.05; Figure 2D and 2E). Glucose supplementation provided an intermediate response, that did not differ from either Control or Hyper-O-GlcNAcylated cells (*p* > 0.05; Figure 2E). Hypo-O-GlcNAcylated cells (OSMI-1-treated) had the least invasion potential, comparable to Controls (*p* > 0.05; Figure 2E and 2F).

### Hyper-O-GlcNAcylation promotes the EMT phenotype

**Figure 3:**
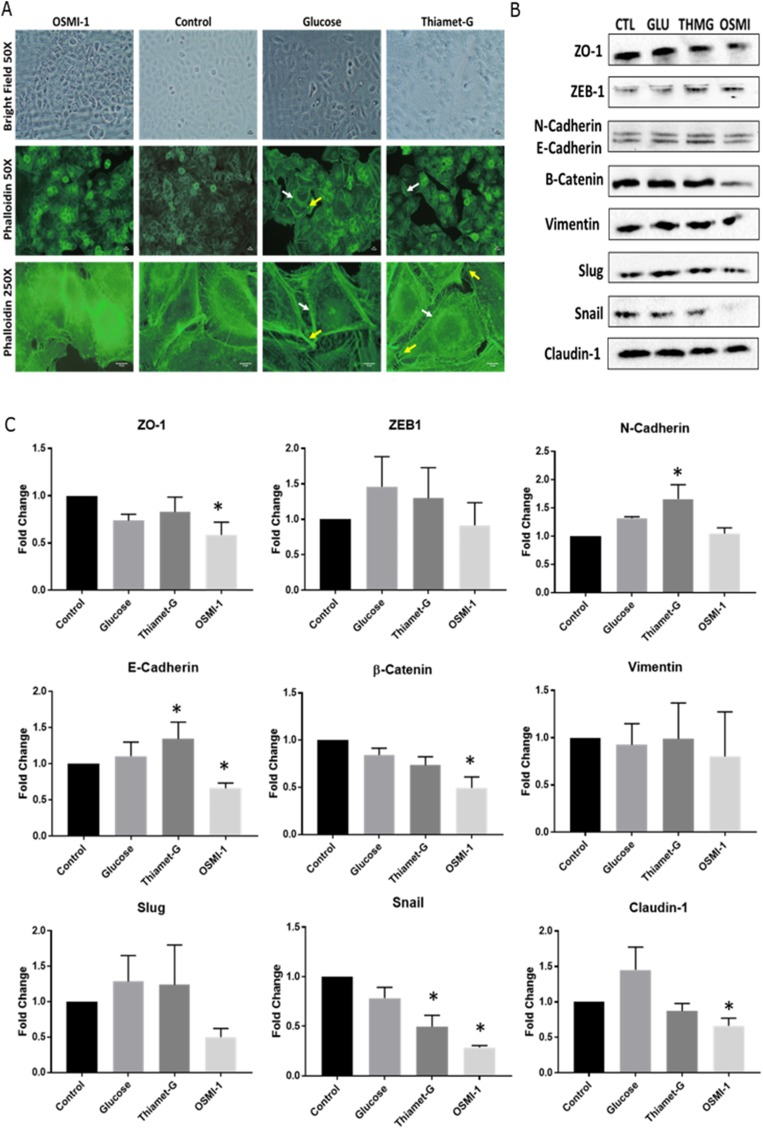
Disruption to O-GlcNAc signaling alters cell morphology and EMT protein expression. (**A**) Representative images of cultured Ishikawa cells treated with OSMI-1, Thiamet-G, 25 mM glucose, or vehicle for 48 hours. Immunofluorescent staining of actin with phalloidin was assessed for stress fibers (white arrows), and focal adhesions (yellow arrows) as indicators of EMT. (**B**) Representative Western Blots (*n* = 6 biological replicates) of whole cell lysates probed for epithelial and mesenchymal protein markers of EMT in Ishikawa cells treated as above for 48 hours. (**C**) Bar graphs depicting the densitometry analysis of the Western Blots. Bars depict the mean +/– SEM (*n* = 6 biological replicates for each protein. An asterisk (^*^) denotes differences compared to control (*p* < 0.05).

Aspects of the EMT process were examined in Ishikawa cells at both the mRNA and protein levels by manipulating O-GlcNAcylation (as described above). Phalloidin staining of stress filaments in the cells (F-actin) revealed diffuse and epithelial-like staining in Hypo-O-GlcNAcylated (OSMI-1- treated) cells (Figure 3A). Conversely, Hyper-O-GlcNAcylated (Glucose and ThmG-treated) cells had conspicuous actin staining throughout the cytoplasm with stress fiber-like bundles along the marginal borders of the cells (white arrows, Figure 3A), as well as focal adhesions (yellow arrows, Figure 3A), indicative of mesenchymal cell morphology.

Despite the above-described morphological changes in Hypo- and Hyper-O-GlcNAcylated cells, there was no evidence that transcript abundance for key EMT markers (*ZEB1*, *CDH1*, *CDH2*, *CTNNB1*, *VIM*, *SNAI1*, *SNAI2*, and *CLDN1*) changed among the treatment groups as assessed by qPCR analysis (Supplementary Table 1). Conversely, immunoblots indicated an increase in E-Cadherin (CDH1) expression in Hyper-O-GlcNAcylated (ThmG-treated) cells and a decrease in Hypo-O-GlcNAcylated (OMSI-1 treated) cells compared to Controls (*P* < 0.05; Figure 3B and 3C). Consistently, expression of the E-Cadherin suppressor, Snail, was reduced by these same treatments (*p* < 0.05; Figure 3C). The expression of β-Catenin was sustained by Hyper-O-GlcNAcylation (ThmG), but down-regulated by Hypo-O-GlcNAcylation (OSMI-1; (*p* < 0.05; Figure 3C). Claudin-1, a major constituent of tight junction complexes, was markedly decreased in Hypo-O-GlcNAcylatied (OSMI-1-treated) cells compared to Controls (*p* < 0.05; Figure 3C). Relative expression of ZO-1 was also reduced by OSMI-1 treatment (*p* < 0.05; Figure 3C). None of the other EMT markers detected (i.e., Vimentin, Slug, ZEB1) were affected by O-GlcNAcylation status (*p* > 0.05; Figure 3C).

### Microarray analysis corroborates dysregulation of O-GlcNAcylation as a mechanism of EMT in endometrial cancer

**Figure 4:**
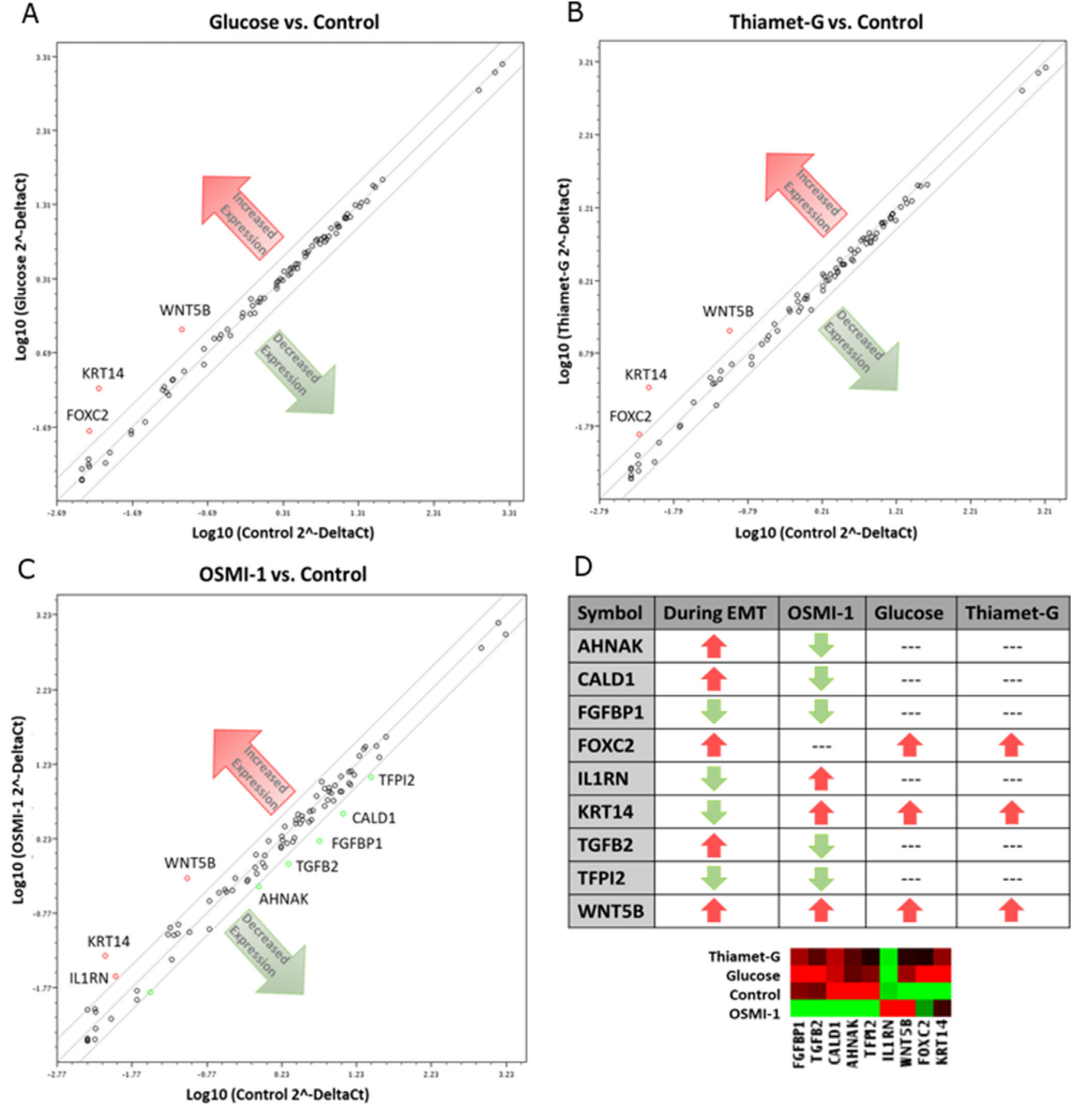
Disruption of O-GlcNAc alters gene expression in EMT related genes of Ishikawa cells. Cells were cultured with OSMI-1, Thiamet-G, 25 mM glucose, or vehicle for 48 hours before harvesting for RNA isolation. The mRNA was the quantified by RT-PCR (*n* = 3 biological replicates) and the relative expression is depicted for cells grown in (**A**) 25 mM glucose, (**B**) Thiamet-G, and (**C**) OSMI-1 compared to vehicle (control). The lines encompass 2.5-fold changes. Genes up-regulated are denoted by red circles, whereas genes down-regulated appear as green circles. Black circles constitute genes relatively unchanged. (**D**) Chart and heatmap summarizing the gene expression results compared to anticipated outcomes for EMT based upon literature review.

The above phenotypic and molecular changes in response to altered O-GlcNAcylation were corroborated by mRNA analysis of 86 genes important to the process of EMT. The EMT RT^2^ Profiler PCR Array and GeneGlobe Data Analysis Center analysis tool (Qiagen), was used to evaluate changes in gene expression (2.5 fold or greater) compared to Control cells (Supplementary Table 2). Three independent experiments yielded an identical mRNA expression profile for both Hyper-O-GlcNAcylation treatments (i.e., Thiamet-G and Glucose), with increased expression of *FOXC2* and *WNT5B*, both promoters of EMT, and *KRT14*, a cytoskeletal intermediate filament monomer (Figure 4A, 4B and 4D). Hypo-O-GlcNAcylated (OSMI-1-treated) cells also had increased *WNT5B* and *KRT14;* however, expression of *AHNAK*, *CALD1*, *FGFBP1*, *TGFB2*, and *TFPI2* genes were decreased (Figure 4C and 4D).

## DISCUSSION

O-GlcNAcylation is a recently discovered post-translational modification of cellular proteins that is highly abundant and ubiquitous throughout the nuclear and cytoplasmic compartments of the cell, yet its influence on cellular processes is poorly understood. First-discovered about 30 years ago [13], O-GlcNAc is an end-product of the hexosamine biosynthesis pathway (HBP), where its relative concentration serves as nutrient sensor indicative of metabolic status [14]. Indeed, elevated O-GlcNAcylation (i.e., Hyper- O-GlcNAcylation), for example, is a hallmark of high metabolism seen in many types of cancers [15], including cancers of the breast [16], liver [17], bladder [18], prostate [19], lung [20], colon [20, 21], and endometrium [3]. Thus, O-GlcNAc, and by extension O-GlcNAcylation, may constitute an important regulator of cancer development and progression, while simultaneously providing a potential, novel connection between the onset of T2D and the increased risk of certain cancers [22]. Endometrial cancers fall into this category of increased risk attributable to T2D [5, 23, 24], and the current study reveals that aberrant O-GlcNAcylation can mechanistically-augment the tumorigenicity of endometrial cancer cells. These findings are consistent with the previous assertion that O-GlcNAc enzyme gene expression is increased along the myometrial invasive edge of endometrial tumors [3]. Similarly, elevated expression of *OGT* and *MGEA5* mRNA is consistent with the nutrient-sensing nature of O-GlcNAcylation. High nutrient intake, hyperglycemia, and other metabolic abnormalities all promote the flow of glucose into the HBP, resulting in elevated O-GlcNAcylation. In this way, the current findings of the meta-analysis add support to the concept that O-GlcNAc modification has a role in the development of diabetic complications and cancer [2, 25, 26].

One mechanism responsible for increased tumorigenicity in many cancers is the phenomenon of epithelial-mesenchymal transition (EMT). Although it is acknowledged that EMT is a critical process of embryogenesis, this same process enhances tumor development and metastasis [27–29]. Some of the key characteristics of EMT include the loss of cell polarity and cell-cell adhesions, and an increase in invasive properties [30, 31]. In the current study, Hyper-O-GlcNAcylation supported cell proliferation, induced reorganization of the cytoskeleton, and promoted migration and invasion of endometrial cancer (Ishikawa) cells. This was evidenced by a significant reduction in cell proliferation at 72 hours of growth compared to hyper and normally O-GlcNAcylated cells when O-GlcNAcylation was reduced by inhibition of OGT, (Figure 2A and 2B). Cytoskeletal reorganization was noted by F-actin staining in hyper-O-GlcNAcylated cells (Glucose and Thiamet-G) by an observable increase in stress fibers and focal adhesions compared to control (DMSO) and hypo-O-GlcNAcylated (OSMI-1) cells (Figure 3A). Results of a matrigel invasion assay provided evidence that increased O-GlcNAcylation by Thiamet-G treatment increased the invasiveness of Ishikawa cells in culture, (Figure 2E and 2F). Similarly, a wound healing assay suggested that reduced O-GlcNAcylation (OSMI-1) impaired cell migration, even when controlling for proliferation (serum free conditions, 48 hours) (Figure 2C and 2D).

Intercellular adhesion, mediated largely by cadherin and catenin proteins, is a critical obstacle for epithelial cells to overcome as they de-differentiate and become more tumorigenic. A decrease in E-Cadherin expression, for instance, is among the initiating steps of EMT [32, 33], and is a notable consequence of Hyper-O-GlcNAcylation in many cell types [34]. Snail1, an E-Cadherin suppressor, is stabilized by O-GlcNAc at Ser112 under hyperglycemic culture conditions [9], and O-GlcNAcylation of E-Cadherin can occur directly via the cytoplasmic domain, which consequently inhibits its transport to the cell surface and prevents intercellular adhesion [35]. In the current study, Hyper-O-GlcNAcylation unexpectedly decreased the expression of Snail1, in turn increasing E-Cadherin expression. However, its mesenchymal counterpart N-Cadherin was also increased by hyper-O-GlcNAcylation. This suggests that the alteration of E-Cadherin expression is perhaps less critical for EMT than that of N-Cadherin in Ishikawa cells, or that the period of time required for this type of transition is much longer than that evaluated in the current experiments (48 hrs).

A major group of cellular signaling pathways associated with EMT are the Wnt signaling pathways. Of the Wnt pathways, 3 have been characterized; the canonical pathway, the noncanonical pathway, and the noncanonical Wnt/Calcium pathway [29]. In the canonical pathway, Wnt induces the expression of the mesenchymal-like protein, Beta-catenin. Beta-catenin accumulates in the cytoplasm and is translocated to the nucleus, where it acts as a transcriptional activator of TCF/LEF family transcription factors to induce a cellular response [36]. A recent study investigating the role of high glucose concentrations in endometrial cancer found that high glucose increased the flux of glucose into the HBP, in turn increasing O-GlcNAcylation of proteins [37]. Hyper-O-GlcNAcylation also increased the expression of B-catenin [37]. In the current study, however, an upregulation of the non-canonical Wnt ligand, WNT5B, by either Hyper- or Hypo-O-GlcNAcylation did not affect the canonical Wnt ligands (i.e. Wnts –1, –2, –3, –8a, –8b, –10a, –10b) [38] or β-catenin expression (Figure 4 and 3B, respectively). The non-canonical Wnt pathways are not as well understood as the canonical, partly due to the diversity and sheer number of pathways. However, one such pathway activated by Wnt5b is the Planar Cell Polarity (PCP) pathway, which controls tissue polarity and cell migration [39]. WNT5B is up-regulated in MCF-7 cells [40] and is associated with cell migration and proliferation in lung cancer cells [41]. In preadipocytes, WNT5B overexpression partially inhibits canonical Wnt/β-catenin signaling, thereby promoting adipogenesis [42]. In addition to its role in cancer and metastasis, non-canonical Wnt signaling also promotes metabolic dysfunction and adipose tissue inflammation, which is critical to the development of insulin resistance [43]. Collectively, these observations lead us to suggest that, in Ishikawa cells, dysregulation of O-GlcNAc signaling could promote EMT through activation of non-canonical Wnt signaling via WNT5B overexpression, which may in turn result in inhibited Wnt/β-catenin signaling. Such a mechanism could also help explain the connection between insulin resistance and increased risk of endometrial cancer.

In addition to increased WNT5B expression, the current study revealed that the transcription factor, Forkhead box protein C2 (FOXC2) is also highly-expressed in Hyper-O-GlcNAcylated Ishikawa cells (Figure 4A, 4B and 4D). Often, increased expression of FOXC2 during EMT is associated with highly-metastatic cancers [44], some of which can be reduced by shRNA therapy [45]. In podocytes, for instance, increased FOXC2 is associated with increased Vimentin expression, cytoskeletal reorganization, disruption to ZO-1 localization, and increased cell motility [46]. In the current study, cytoskeletal re-organization was noted in hyper-O-GlcNAcylated cells (Figure 3A), and although Vimentin expression was consistent among treatments, ZO-1 expression was reduced by inhibition of OGT (hypo-O-GlcNAcylation, OSMI-1) (Figure 3C). Thus, there is support for the concept that Hyper-O-GlcNAcylation of Ishikawa cells induces EMT and metastasis via a FOXC2-mediated mechanism.

Cytoskeletal re-organization is key feature of EMT. Cytokeratin intermediate filaments are often considered biomarkers of cancers, but are more recently gaining attention as mediators of disease [47, 48]. A good example of this is Keratin 18, which is a component of cytokeratin 8/18 intermediate filaments and has long been used as a marker for apoptosis in cancers [49], but the loss of keratin 18 expression in cells is associated with EMT [50]. Previously we have determined that Hypo-O-GlcNAcylation in cervical cancer cells reduces and re-organizes cytokeratin 8/18 filaments, suggesting that O-GlcNAcylation influences filament expression and stability [51]. Similar restructuring of filaments in Ishikawa cells occurred in the current study, with both Hyper- and Hypo-O-GlcNAcylation increasing the overall expression of Keratin 14 mRNA. Co-expression of Vimentin and keratin in MCF-7 breast cancer cells increases cell invasiveness [52]. In the current study, Hyper-O-GlcNAcylation had no effect on Vimentin expression, increased Keratin 14 expression, and yet still increased metastatic potential. These findings warrant further investigation into the regulation of keratin filaments by O-GlcNAcylation, and their possible role in disease progression.

In summary, dysregulation of O-GlcNAcylation supports EMT and cytoskeletal re-organization in endometrial cancer cells, potentially through activation of the noncanonical Wnt signaling pathway via WNT5B. Additionally, Hyper-O-GlcNAcylation induces the expression of FOXC2 and augments the invasion potential of the cells. These findings indicate Hyper-O-GlcNAcylation, such as that observed in diabetic individuals [53], could enhance the aggressiveness of endometrial cancer. Conversely, Hypo-O-GlcNAcylation impaired endometrial cancer cell proliferation and wound healing, and down-regulated expression of pro-EMT genes (AHNAK, CALD1, and TGFB2). These observations suggest that metabolic status, particularly as it relates to O-GlcNAcylation within cells, might be an important consideration in therapeutic approaches to endometrial cancer. Future studies should focus on aberrant O-GlcNAcylation as a potential marker for more aggressive disease in human tissues, and the inhibition of Hyper-O-GlcNAcylation as a potential treatment in diabetic patients.

## MATERIALS AND METHODS

### Cell culture/Reagents

Endometrial Cancer cells (Ishikawa) were obtained from Sigma Aldrich (cat. #99040201). Cell Line authentication was performed before, during, and after experimentation in the University of Vermont Cancer Center Advanced Genome Technologies Core and was supported by the University of Vermont Cancer Center, Lake Champlain Cancer Research Organization, and the University of Vermont College of Medicine. O-GlcNAcylation in cells was manipulated by the OGA inhibitor, ThmG (1 μM, Fisher Scientific), the OGT inhibitor, OSMI-1 (50 μM, Aobious), and supplementation of excess glucose (25 mM). ThmG and OSMI-1 were dissolved in DMSO (Fisher Scientific) at a concentration of 1000X, thus control and glucose treated cells received 0.1% DMSO (vehicle). Cell culture media (EMEM, 10% FBS and 10 μl/mL antibiotic-anti-mycotic (Fisher Scientific) was exchanged every 24 hours unless otherwise specified.

### Immunoblotting

Cell Lysates were harvested via trypsin digestion and dissolved in RIPA buffer, and then passaged 5 times through a 26G needle. Protein concentration was measured by BCA Assay (BioRad) following the manufacturer’s protocol. Pre-stained SDS-PAGE gels were loaded with 20 μg of proteins per lane. Protein was then transferred to PVDF membrane (Millipore). Membranes were then probed with antibodies associated with EMT (EMT antibody kit), O-GlcNAc (CTD 110.6) (Supplementary Figure 1), and OGT. Goat-anti-rabbit and goat-anti-mouse HRP conjugated secondary antibodies were used in combination with Clarity Western ECL Blotting Substrate (BioRad) for imaging on the BioRad ChemiDoc Imager. All antibodies were purchased from Cell Signaling unless otherwise specified.

### Immunohistochemistry

Cells were seeded on glass coverslips and incubated in treatments described above for 72 hours. Media was exchanged daily. Cells were then fixed with 4% paraformaldehyde in PBS (Fisher Scientific) and stained with Alexaflour-488 Phalloidin (Molecular Probes, Eugene, OR) and DAPI (Molecular Probes). Cells were imaged with the Olympus CKX53 at 200–1000X.

### Proliferation

In a set of 3 independent experiments, cells were seeded in quadruplicate in flat-sided Thermo Fisher Scientific™ Nunc™ Cell Culture Tubes at an initial seeding density of 50 k cells/ml of serum free culture medium. Cells were treated as described above. The conditioned culture medium was exchanged daily, and the cells for each group were harvested for counting at 24-hour intervals over a four-day period. An MTS assay was also used to measure proliferation and viability. Clear 96 well plates were seeded with 5,000 cells/well in serum free conditions. Cells were treated as described above, and conditioned medium was refreshed daily. Cell proliferation was measured with the CellTiter 96^®^ AQueous One Solution Cell Proliferation Assay (MTS) kit following manufacturer instructions (Promega, Madison, WI) at 24 and 48 hours as measured by the Synergy HT Plate Reader (Biotek, Winooski, VT).

### Migration

A wound-healing assay was used to measure migration of cells treated as described. Confluent monolayers of treated cells grown in 24 well plates were serum starved for 24 hours before “wounds” were created by running a 10 μL pipette tip across the monolayer. Cells were maintained in serum free conditions to inhibit proliferation and were incubated at 37°C and 5% CO_2_ for 48 hours. Images of the “wound” area were taken every 24 hours (100X) and the “wound” area was measured with ImageJ software to calculate percent wound closure.

### Invasion

Corning Matrigel Biocoat Invasion Chambers (24 well, 0.8 μ) (Corning, Bedford, MA) were used to assess the invasion potential of treated cells compared to control. The upper chamber was seeded with 100,000 serum-starved cells. Cells were exposed to O-GlcNAc-modifying treatments at the time of seeding. The lower chamber contained 5% FBS as a chemoattractant. Cells were incubated for 48 hours at 37°C and 5% CO_2_. Cells were then removed from the upper chamber and the Transwell membranes were fixed with 100% methanol and stained with Crystal Violet. Invaded cells were then counted by two independent researchers and the results were averaged.

### RT PCR array

RNA was extracted from treated cell pellets with the RNeasy Mini-Kit (Qiagen) with DNase digestion (RNAse free DNase Kit, QIAGEN) following the manufacturer protocols. The cDNA synthesis and RT^2^ Profiler PCR Arrays (QIAGEN, PAHS-090Z) (Supplementary Table 2) were performed in the University of Vermont Cancer Center Advanced Genome Technologies Core and was supported by the University of Vermont Cancer Center, Lake Champlain Cancer Research Organization, and the University of Vermont College of Medicine. Results were analyzed via the GeneGlobe Data Analysis Center (QIAGEN) with 2.5 fold change serving as the cut-off.

### Oncogenomic data analysis

Multidimensional cancer genomic data analysis was performed using online data-mining tool from the cBioPortal for Cancer Genomics (http://cbioportal.org) and the data sets from the cBioPortal for Cancer Genomics and the TCGA research Network (http://cancergenome.nih.gov) [54, 55]. Tumor types and data sets are chosen in accordance with the publication guidelines from TCGA (last update:30 September 2014). Genomic alterations were identified when the following occurred: (1) gene mutations; (2) putative copy number alteration (amplification or deletion); (3) RNA expression Z-scores (RNA Seq Version 2 RSEM) with Z-score thresholds ± 2.0; and (4) protein/phospho-protein level (RPPA) with Z-score thresholds ± 2.0.

### Data analysis

A minimum of 3 independent replicates were completed for each experiment, reported as mean +/– SEMs. Statistical analysis was conducted using 1 or 2-Way ANOVA and Student’s *t*-tests as appropriate with Graph Pad Prism Statistical Analysis Software. Fold-change differences in mRNA expression were calculated with the GeneGlobe Data Analysis Center (QIAGEN) for RT^2^ Profiler PCR Arrays.

## SUPPLEMENTARY MATERIALS



## Abbreviations

O-GlcNAc: β-N-acetylglucosamine
OGT: O-GlcNAc Transferase
OGA: O-GlcNAcase
HBP: Hexosamine Biosynthesis Pathway
T2D: Type 2 Diabetes
ThmG: Thiamet-G
FBS: Fetal Bovine Serum
