# Supplementary material for: Hyper-O-GlcNAcylation promotes epithelial-mesenchymal transition in endometrial cancer cells
Source: Oncotarget. 2019 Apr 23;10(30):2899–910. doi: 10.18632/oncotarget.26884 (PMC6499600; doi:10.18632/oncotarget.26884)
Supplement: Supplementary file 1 [file oncotarget-10-2899-s001.pdf]

Hyper-O-GlcNAcylation promotes epithelial-mesenchymal transition in endometrial cancer cells

SUPPLEMENTARY MATERIALS

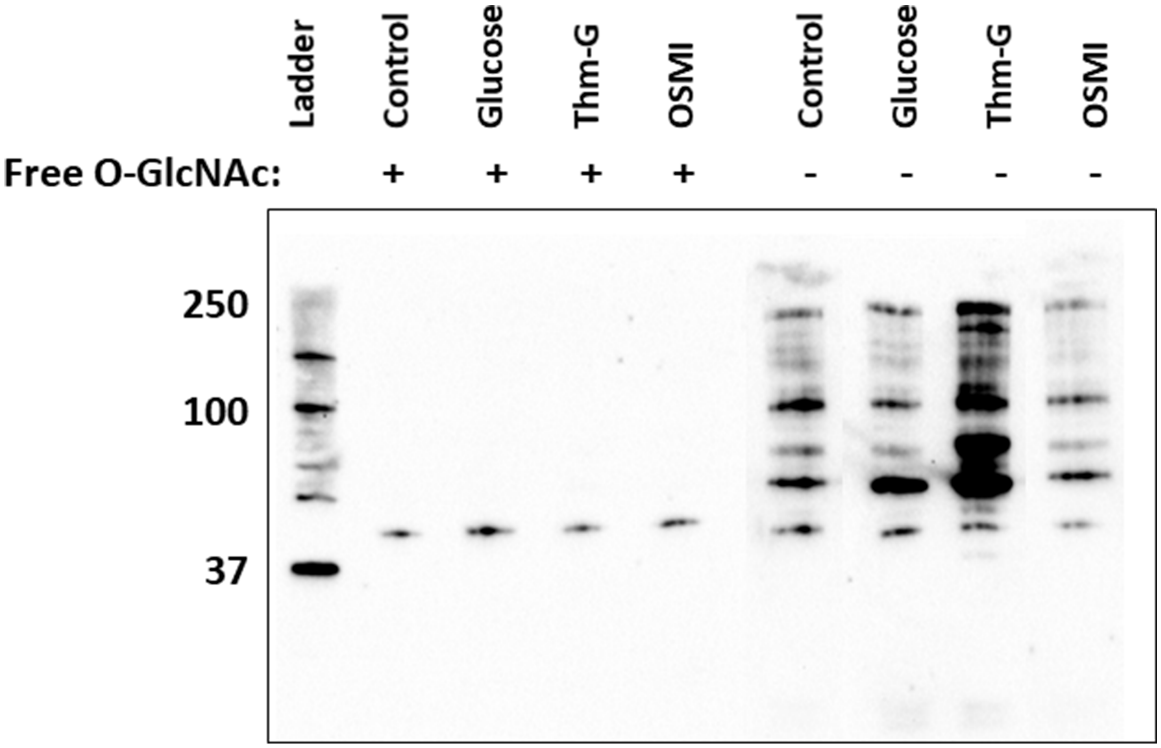

Supplementary Figure 1: Verification of O-GlcNAc (CTD110.6) mouse mAb specificity by Western blot. Whole cell lysates (treated with Vehicle (DMSO), 25 mM Glucose, 1 uM ThiametG, or 50 µM OSMI-1) were incubated in O-GlcNAc (CTD110.6) Mouse mAb (1:2,500 in TBST, 5% w/v BSA) without or with 100 mM Free N-acetylglucosamine (Free O-GlcNAc) as described by Zachara, et al. Curr Protoc Mol Biol. 2011 Chapter 17: Unit 17.6 As seen in the samples exposed to free O-GlcNAc, non-specific binding of the primary antibody was minimal.

Supplementary Table 1: RT[2] PCR Profiler PCR Array (catalog# PAHS-090Z, QIAGEN) results depicted in plate set up, these results are also depicted in Figure 4

25mM Glucose vs. Control (fold change)

Layout	01	02	03	04	05	06	07	08	09	10	11	12
A	AHNAK<br>1.00<br>C	AKT1<br>-1.02	BMP1<br>1.09	BMP2<br>1.21<br>B	BMP7<br>1.00<br>C	CALD1<br>1.04	CAMK2N1<br>1.44<br>B	CAV2<br>1.24	CDH1<br>1.19	CDH2<br>1.23	COL1A2<br>1.19	COL3A1<br>1.44
B	COL5A2<br>-1.01<br>B	CTNNB1<br>-1.10	DSC2<br>-1.00	DSP<br>1.20	EGFR<br>1.20	ERBB3<br>1.13	ESR1<br>1.03	F11R<br>1.15	FGFBP1<br>1.35	FN1<br>1.18	FOXC2<br>3.67<br>B	FZD7<br>1.58
C	GNG11<br>1.32	GSC<br>1.00<br>C	GSK3B<br>1.16	IGFBP4<br>1.43<br>B	IL1RN<br>1.01<br>B	ILK<br>1.06	ITGA5<br>1.20	ITGAV<br>1.18	ITGB1<br>1.16	JAG1<br>1.17	KRT14<br>10.19<br>B	KRT19<br>1.15
D	KRT7<br>1.16<br>B	MAP1B<br>1.09	MMP2<br>-1.14<br>B	MMP3<br>1.00<br>C	MMP9<br>-1.06<br>B	MSN<br>1.05	MST1R<br>1.20	NODAL<br>1.02<br>B	NOTCH1<br>1.07	NUDT13<br>1.31	OCLN<br>1.35	PDGFRB<br>1.03<br>B
E	PLEK2<br>1.24	DESI1<br>-1.01	PTK2<br>1.12	PTP4A1<br>1.08	RAC1<br>1.29	RGS2<br>-1.05	SERPINE1<br>1.40	GEMIN2<br>1.21	SMAD2<br>1.25	SNAI1<br>1.30<br>B	SNAI2<br>-1.19<br>B	SNAI3<br>-1.14
F	SOX10<br>1.33<br>B	SPARC<br>1.57<br>B	SPP1<br>1.02	STAT3<br>1.04	STEAP1<br>1.19	TCF3<br>1.06	TCF4<br>1.09	TFPI2<br>-1.04	TGFB1<br>1.01	TGFB2<br>1.38	TGFB3<br>1.29	TIMP1<br>1.11
G	TMEFF1<br>1.21	TMEM132<br>A<br>1.02	TSPAN13<br>1.10	TWIST1<br>1.12	VCAN<br>1.27	VIM<br>1.21	VPS13A<br>1.29	WNT11<br>1.40<br>B	WNT5A<br>1.26	WNT5B<br>5.06<br>A	ZEB1<br>1.41<br>B	ZEB2<br>1.42<br>B

Thiamet-G vs. Control (fold change)

Layout	01	02	03	04	05	06	07	08	09	10	11	12
A	AHNAK<br>-1.38<br>C	AKT1<br>-1.47	BMP1<br>-1.19	BMP2<br>-1.37<br>B	BMP7<br>-1.04<br>B	CALD1<br>-1.09	CAMK2N1<br>1.16<br>B	CAV2<br>-1.02	CDH1<br>-1.13	CDH2<br>-1.25	COL1A2<br>-1.38	COL3A1<br>-1.13
B	COL5A2<br>-1.94<br>B	CTNNB1<br>-1.01	DSC2<br>1.18	DSP<br>-1.07	EGFR<br>-1.10	ERBB3<br>1.13	ESR1<br>-1.18	F11R<br>-1.12	FGFBP1<br>1.07	FN1<br>-1.00	FOXC2<br>2.32<br>B	FZD7<br>1.11
C	GNG11<br>1.13	GSC<br>-1.38<br>C	GSK3B<br>-1.27	IGFBP4<br>1.05<br>B	IL1RN<br>-1.04<br>B	ILK<br>-1.19	ITGA5<br>1.06	ITGAV<br>1.06	ITGB1<br>-1.26	JAG1<br>-1.06	KRT14<br>7.53<br>B	KRT19<br>-1.06
D	KRT7<br>1.11<br>B	MAP1B<br>-1.77	MMP2<br>1.16<br>B	MMP3<br>1.02<br>B	MMP9<br>-1.21<br>B	MSN<br>-1.06	MST1R<br>-1.16	NODAL<br>-1.16<br>B	NOTCH1<br>-1.28	NUDT13<br>-1.09	OCLN<br>1.06	PDGFRB<br>-1.38<br>C
E	PLEK2<br>-1.10	DESI1<br>-1.37	PTK2<br>-1.26	PTP4A1<br>-1.18	RAC1<br>1.06	RGS2<br>-1.21	SERPINE1<br>-1.25	GEMIN2<br>-1.14	SMAD2<br>-1.20	SNAI1<br>1.16<br>A	SNAI2<br>-1.68<br>B	SNAI3<br>-1.56
F	SOX10<br>-1.10<br>B	SPARC<br>1.24<br>B	SPP1<br>1.38	STAT3<br>-1.33	STEAP1<br>1.13	TCF3<br>-1.21	TCF4<br>-1.22	TFPI2<br>-1.31	TGFB1<br>-1.20	TGFB2<br>-1.03	TGFB3<br>1.03	TIMP1<br>1.01
G	TMEFF1<br>-1.14	TMEM132<br>A<br>-1.15	TSPAN13<br>-1.18	TWIST1<br>-1.16	VCAN<br>1.13	VIM<br>-1.40	VPS13A<br>-1.15	WNT11<br>1.32<br>B	WNT5A<br>-1.29	WNT5B<br>3.58<br>A	ZEB1<br>1.48<br>B	ZEB2<br>-1.38<br>C

OSMI-1 vs. Control (fold change)

Layout	01	02	03	04	05	06	07	08	09	10	11	12
A	AHNAK<br>-1.11<br>C	AKT1<br>-1.09	BMP1<br>-1.14	BMP2<br>1.04<br>B	BMP7<br>-1.05<br>B	CALD1<br>-2.53	CAMK2N1<br>2.41<br>A	CAV2<br>-1.19	CDH1<br>1.41	CDH2<br>1.37	COL1A2<br>-1.16	COL3A1<br>1.56
B	COL5A2<br>1.67<br>B	CTNNB1<br>-1.18	DSC2<br>1.30	DSP<br>1.14	EGFR<br>1.08	ERBB3<br>1.55	ESR1<br>1.26	F11R<br>1.17	FGFBP1<br>-2.88	FN1<br>1.04	FOXC2<br>1.75<br>B	FZD7<br>1.61
C	GNG11<br>-1.15	GSC<br>-1.11<br>C	GSK3B<br>1.35	IGFBP4<br>1.58<br>B	IL1RN<br>2.82<br>B	ILK<br>-1.26	ITGA5<br>1.11	ITGAV<br>-1.04	ITGB1<br>1.12	JAG1<br>1.33	KRT14<br>7.35<br>B	KRT19<br>-1.37
D	KRT7<br>-1.20<br>B	MAP1B<br>1.65	MMP2<br>-1.70<br>B	MMP3<br>2.36<br>B	MMP9<br>-1.04<br>B	MSN<br>-1.32	MST1R<br>1.43	NODAL<br>-1.42<br>B	NOTCH1<br>-1.28	NUDT13<br>1.27	OCLN<br>2.03	PDGFRB<br>-1.11<br>C
E	PLEK2<br>-1.18	DESI1<br>1.18	PTK2<br>1.18	PTP4A1<br>1.54	RAC1<br>1.12	RGS2<br>1.45	SERPINE1<br>-1.46<br>A	GEMIN2<br>1.09	SMAD2<br>1.20	SNAI1<br>1.14<br>B	SNAI2<br>-1.14<br>B	SNAI3<br>1.65
F	SOX10<br>-1.30<br>B	SPARC<br>1.98<br>B	SPP1<br>1.12	STAT3<br>-1.25	STEAP1<br>-1.06	TCF3<br>-1.15	TCF4<br>-1.04	TFPI2<br>-1.94	TGFB1<br>1.08	TGFB2<br>-2.23	TGFB3<br>-1.06	TIMP1<br>1.21
G	TMEFF1<br>1.60	TMEM132<br>A<br>1.31	TSPAN13<br>-1.20	TWIST1<br>1.14	VCAN<br>1.11	VIM<br>-1.07	VPS13A<br>1.52	WNT11<br>2.03<br>A	WNT5A<br>-1.14	WNT5B<br>6.49<br>A	ZEB1<br>2.07<br>B	ZEB2<br>-1.03<br>B

Supplementary Table 2: Gene table for RT² PCR Profiler PCR Array catalog# PAHS-090Z

Position	Symbol	Description
A01	AHNAK	AHNAK nucleoprotein
A02	AKT1	V-akt murine thymoma viral oncogene homolog 1
A03	BMP1	Bone morphogenetic protein 1
A04	BMP2	Bone morphogenetic protein 2
A05	BMP7	Bone morphogenetic protein 7
A06	CALD1	Caldesmon 1
A07	CAMK2N1	Calcium/calmodulin-dependent protein kinase II inhibitor 1
A08	CAV2	Caveolin 2
A09	CDH1	Cadherin 1, type 1, E-cadherin (epithelial)
A10	CDH2	Cadherin 2, type 1, N-cadherin (neuronal)
A11	COL1A2	Collagen, type I, alpha 2
A12	COL3A1	Collagen, type III, alpha 1
B01	COL5A2	Collagen, type V, alpha 2
B02	CTNNB1	Catenin (cadherin-associated protein), beta 1, 88kDa
B03	DSC2	Desmocollin 2
B04	DSP	Desmoplakin
B05	EGFR	Epidermal growth factor receptor
B06	ERBB3	V-erb-b2 erythroblastic leukemia viral oncogene homolog 3 (avian)
B07	ESR1	Estrogen receptor 1
B08	F11R	F11 receptor
B09	FGFBP1	Fibroblast growth factor binding protein 1
B10	FN1	Fibronectin 1
B11	FOXC2	Forkhead box C2 (MFH-1, mesenchyme forkhead 1)
B12	FZD7	Frizzled family receptor 7
C01	GNG11	Guanine nucleotide binding protein (G protein), gamma 11
C02	GSC	Goosecoid homeobox
C03	GSK3B	Glycogen synthase kinase 3 beta
C04	IGFBP4	Insulin-like growth factor binding protein 4
C05	IL1RN	Interleukin 1 receptor antagonist
C06	ILK	Integrin-linked kinase
C07	ITGA5	Integrin, alpha 5 (fibronectin receptor, alpha polypeptide)
C08	ITGAV	Integrin, alpha V (vitronectin receptor, alpha polypeptide, antigen CD51)
C09	ITGB1	Integrin, beta 1 (fibronectin receptor, beta polypeptide, antigen CD29 includes MDF2, MSK12)
C10	JAG1	Jagged 1
C11	KRT14	Keratin 14
C12	KRT19	Keratin 19
D01	KRT7	Keratin 7
D02	MAP1B	Microtubule-associated protein 1B
D03	MMP2	Matrix metallopeptidase 2 (gelatinase A, 72kDa gelatinase, 72kDa type IV collagenase)
D04	MMP3	Matrix metallopeptidase 3 (stromelysin 1, progelatinase)
D05	MMP9	Matrix metallopeptidase 9 (gelatinase B, 92kDa gelatinase, 92kDa type IV collagenase)
D06	MSN	Moesin
D07	MST1R	Macrophage stimulating 1 receptor (c-met-related tyrosine kinase)
D08	NODAL	Nodal homolog (mouse)
D09	NOTCH1	Notch 1
D10	NUDT13	Nudix (nucleoside diphosphate linked moiety X)-type motif 13
D11	OCLN	Occludin
D12	PDGFRB	Platelet-derived growth factor receptor, beta polypeptide
E01	PLEK2	Pleckstrin 2
E02	DESI1	PPPDE peptidase domain containing 2
E03	PTK2	PTK2 protein tyrosine kinase 2

E04	PTP4A1	Protein tyrosine phosphatase type IVA, member 1
E05	RAC1	Ras-related C3 botulinum toxin substrate 1 (rho family, small GTP binding protein Rac1)
E06	RGS2	Regulator of G-protein signaling 2, 24kDa
E07	SERPINE1	Serpin peptidase inhibitor, clade E (nexin, plasminogen activator inhibitor type 1), member 1
E08	GEMIN2	Survival of motor neuron protein interacting protein 1
E09	SMAD2	SMAD family member 2
E10	SNAI1	Snail homolog 1 (Drosophila)
E11	SNAI2	Snail homolog 2 (Drosophila)
E12	SNAI3	Snail homolog 3 (Drosophila)
F01	SOX10	SRY (sex determining region Y)-box 10
F02	SPARC	Secreted protein, acidic, cysteine-rich (osteonectin)
F03	SPP1	Secreted phosphoprotein 1
F04	STAT3	Signal transducer and activator of transcription 3 (acute-phase response factor)
F05	STEAP1	Six transmembrane epithelial antigen of the prostate 1
F06	TCF3	Transcription factor 3 (E2A immunoglobulin enhancer binding factors E12/E47)
F07	TCF4	Transcription factor 4
F08	TFPI2	Tissue factor pathway inhibitor 2
F09	TGFB1	Transforming growth factor, beta 1
F10	TGFB2	Transforming growth factor, beta 2
F11	TGFB3	Transforming growth factor, beta 3
F12	TIMP1	TIMP metallopeptidase inhibitor 1
G01	TMEFF1	Transmembrane protein with EGF-like and two follistatin-like domains 1
G02	TMEM132A	Transmembrane protein 132A
G03	TSPAN13	Tetraspanin 13
G04	TWIST1	Twist homolog 1 (Drosophila)
G05	VCAN	Versican
G06	VIM	Vimentin
G07	VPS13A	Vacuolar protein sorting 13 homolog A (S. cerevisiae)
G08	WNT11	Wingless-type MMTV integration site family, member 11
G09	WNT5A	Wingless-type MMTV integration site family, member 5A
G10	WNT5B	Wingless-type MMTV integration site family, member 5B
G11	ZEB1	Zinc finger E-box binding homeobox 1
G12	ZEB2	Zinc finger E-box binding homeobox 2
H01	ACTB	Actin, beta
H02	B2M	Beta-2-microglobulin
H03	GAPDH	Glyceraldehyde-3-phosphate dehydrogenase
H04	HPRT1	Hypoxanthine phosphoribosyltransferase 1
H05	RPLP0	Ribosomal protein, large, P0
H06	HGDC	Human Genomic DNA Contamination
H07	RTC	Reverse Transcription Control
H08	RTC	Reverse Transcription Control
H09	RTC	Reverse Transcription Control
H10	PPC	Positive PCR Control
H11	PPC	Positive PCR Control
H12	PPC	Positive PCR Control
